# Post-Translational Incorporation of L-Phenylalanine into the C-Terminus of α-Tubulin as a Possible Cause of Neuronal Dysfunction

**DOI:** 10.1038/srep38140

**Published:** 2016-12-01

**Authors:** Yanina Ditamo, Yanela M. Dentesano, Silvia A. Purro, Carlos A. Arce, C. Gastón Bisig

**Affiliations:** 1 Centro de Investigaciones en Química Biológica de Córdoba, CIQUIBIC-CONICET, and Departamento de Química Biológica, Facultad de Ciencias Químicas, Universidad Nacional de Córdoba, Ciudad Universitaria, X5000HUA, Córdoba, Argentina

## Abstract

α-Tubulin C-terminus undergoes post-translational, cyclic tyrosination/detyrosination, and L-Phenylalanine (Phe) can be incorporated in place of tyrosine. Using cultured mouse brain-derived cells and an antibody specific to Phe-tubulin, we showed that: (i) Phe incorporation into tubulin is reversible; (ii) such incorporation is not due to *de novo* synthesis; (iii) the proportion of modified tubulin is significant; (iv) Phe incorporation reduces cell proliferation without affecting cell viability; (v) the rate of neurite retraction declines as level of C-terminal Phe incorporation increases; (vi) this inhibitory effect of Phe on neurite retraction is blocked by the co-presence of tyrosine; (vii) microtubule dynamics is reduced when Phe-tubulin level in cells is high as a result of exogenous Phe addition and returns to normal values when Phe is removed; moreover, microtubule dynamics is also reduced when Phe-tubulin is expressed (plasmid transfection). It is known that Phe levels are greatly elevated in blood of phenylketonuria (PKU) patients. The molecular mechanism underlying the brain dysfunction characteristic of PKU is unknown. Beyond the differences between human and mouse cells, it is conceivable the possibility that Phe incorporation into tubulin is the first event (or among the initial events) in the molecular pathways leading to brain dysfunctions that characterize PKU.

Microtubules are filamentous structures found in all eukaryotic cells that run through the entire cytoplasm and are composed mainly of tubulin, a dimeric protein formed by α- and β-subunits. Many important cellular functions involve microtubules. Correct structure and kinetic properties of microtubules are essential for a series of neuronal processes during brain development: mitosis, neuronal architecture, correct guidance, pruning, synapsis, and temporally and spatially regulated delivery of various cargos transported by molecular motors (*e.g.*, myosins, dyneins, kinesins)^1^,^2^,^3^. The numerous and finely regulated biochemical events that underlie these complex processes are controlled by nuclear and external signals. Post-translational modifications of the tubulin protein are one type of regulatory factor. During the 1970s, we described post-translational incorporation of tyrosine (Tyr) into the C-terminus of the tubulin α-subunit^4^,^5^. The α-tubulin chain is biosynthesized with Tyr as the C-terminal amino acid. Following its association with the β-subunit and assembly into microtubules, Tyr is removed by tubulin carboxypeptidase (TCP), resulting in production of a tubulin isospecies termed Glu-tubulin (or detyrosinated tubulin) through exposure of glutamic acid (Glu) as the C-terminal amino acid. As part of a microtubule/tubulin equilibrium process, Glu-tubulin passes to a non-assembled pool where it is rapidly re-tyrosinated by tubulin tyrosine ligase (TTL). This post-translational modification of tubulin is termed the “tyrosination/detyrosination cycle”^6^. Various Tyr analogues, including L-phenylalanine (Phe), L-3,4-dihydroxyphenylalanine (L-Dopa)^7^, 3-Nitro-tyrosine^8^, and azatyrosine^9^ can be incorporated into tubulin in place of Tyr at the same position.

In phenylketonuria (PKU) patients, Phe concentration in blood is higher than normal by a factor of 20–40^10^,^11^. This increase results from a lack of phenylalanine hydroxylase activity or, in some cases, of the main enzyme cofactor. PKU results in abnormal brain development and mental retardation unless the newborn is treated promptly with a low-Phe diet^12^. In a rat experimental model of PKU, Rodriguez and Borisy^13^ found that the percentage of brain tubulin with Phe as the C-terminal amino acid was ~30%, whereas the percentage in control animals was ~3%. This finding suggested that this tubulin modification is related to genesis of mental retardation. However, no studies to date have examined the relationship between this tubulin modification and brain dysfunction observed in human PKU patients.

The present study was focused on the effects of Phe incorporation into the α-tubulin C-terminus on various cellular parameters possibly associated with brain dysfunction in PKU patients. We used a mouse neuron-derived cell line (CAD) as an experimental system, and a newly generated antibody specific to phenylalaninated tubulin (Phe-tubulin). CAD cells proliferate when serum is present in culture medium, and differentiate (emitting neurites) when serum is removed. Re-addition of serum to differentiated cells induces rapid neurite retraction^14^. We observed that Phe incorporation into the α-tubulin C-terminus was associated with: (i) reduced cell proliferation rate; (ii) inhibition of serum-induced retraction of neurites of cultured cells (the presence of Tyr counteracted this effect); (iii) reduced microtubule dynamics at growth cones.

## Results

### Specificity of anti-Phe-tubulin antibody

In our previous studies of Phe incorporation into the α-tubulin C-terminus, we used soluble extracts from rat brain and radiolabeled Phe^15^. For our present purposes, radiolabeled Phe was not useful for study of Phe incorporation into tubulin in living cells because of its insufficient specific radioactivity and the low amount of tubulin in cultured cells. Instead, an antibody specific to Phe-tubulin was needed. We utilized an immunization protocol (described in Supplementary Material) that successfully produced rabbit antisera against other antigens in previous studies. An antiserum (antibody) specific to Phe-tubulin (Suppl. Fig. 1) was thus generated. Soluble rat brain extract (**lane SN**) was treated with carboxypeptidase A to remove all C-terminal Tyr, the enzyme was inhibited, and two separate aliquots were then incubated to incorporate Tyr or Phe into the α-tubulin C-terminus. Aliquots of preparations containing Tyr-tubulin (**lanes 1**) or Phe-tubulin (**lanes 2**) were subjected to Western blotting. Both preparations were positively stained by anti-Total-tubulin antibody. Lanes 1 were stained by anti-Tyr-tubulin antibody but not by anti-Phe-tubulin antibody; the reverse was true for lanes 2. Staining was inhibited by preincubation of the anti-Phe-tubulin antibody with free Phe, but not with free Tyr. Three tubulin isospecies, Tyr-, Glu-, and Δ2-tubulin, are present in soluble brain extracts. Our new anti-Phe-tubulin antibody is therefore highly specific; *i.e.*, it recognizes Phe-tubulin but not Tyr-, Glu-, or Δ2-tubulin, nor any other protein present in soluble rat brain extract.

### Phe is incorporated into tubulin in CAD cells

**Figure 1:**
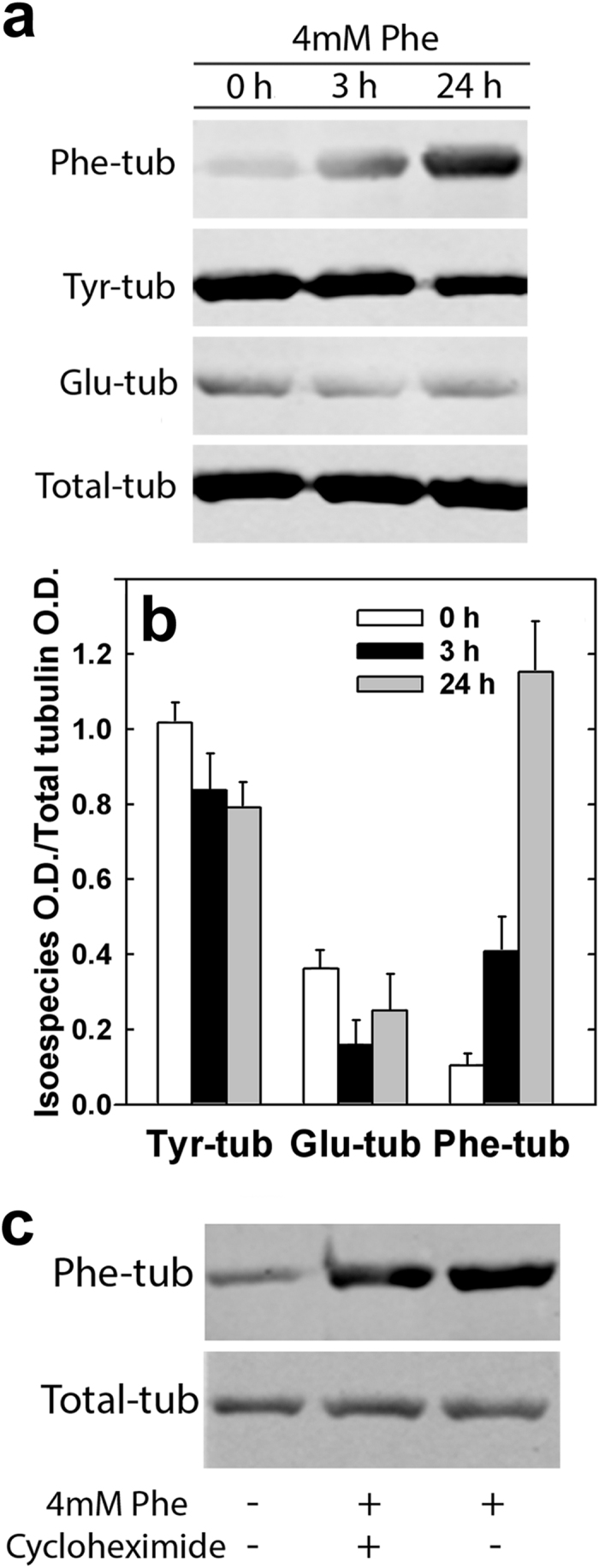
Post-translational incorporation of Phe into tubulin in CAD cells. Cells were cultured 0, 3, or 24 h in medium containing 4 mM Phe, and then analyzed by Western blotting with antibodies directed to Phe-tubulin (Phe-tub), Tyr-tubulin (Tyr-tub), Glu-tubulin (Glu-tub), and Total-tubulin (Total-tub). (**a**) Representative blots. (**b**) Optical density of each tubulin isospecies was standardized relative to Total-tubulin. Data shown are mean ± SD. (**c**) Cells were incubated 3 h in the presence (+) or absence (−) of 4 mM Phe and 100 μM cycloheximide, and analyzed by Western blotting.

To evaluate Phe incorporation into tubulin in living cells, CAD cells were cultured in the absence or presence of 4 mM Phe for 3 or 24 h, and subjected to Western blotting and staining with antibodies directed to Total-, Tyr-, Glu-, and Phe-tubulin. In direct visualization of Western blots, Phe-tubulin band (only faintly visible at time 0) increased as a function of incubation time, whereas Tyr- and Glu-tubulin bands decreased (Fig. 1a). This finding was confirmed by measurement of the optical density of tubulin isospecies standardized to Total-tubulin. At 24 h, Phe-tubulin had increased ~12-fold, whereas Tyr- and Glu-tubulin had decreased ~20% and ~40%, respectively (Fig. 1b). This Phe incorporation into CAD cell tubulin was not blocked by cycloheximide (Fig. 1c), indicating that the presence of Phe in the tubulin C-terminus does not result from *de novo* tubulin biosynthesis but rather from the post-translational mechanism described above. Based on results of Western blotting and densitometry measurements, we constructed standard curves for Total-, Tyr- and Glu-tubulin. Phe-tubulin content in CAD cells after 48 h incubation with 4 mM Phe was 46 ± 4%of Total-tubulin content, and 4 ± 1% of control cell value (Suppl. Fig. 2; Suppl. Table 1).

### Post-translational incorporation of Phe into α-tubulin C-terminus is reversible

**Figure 2:**
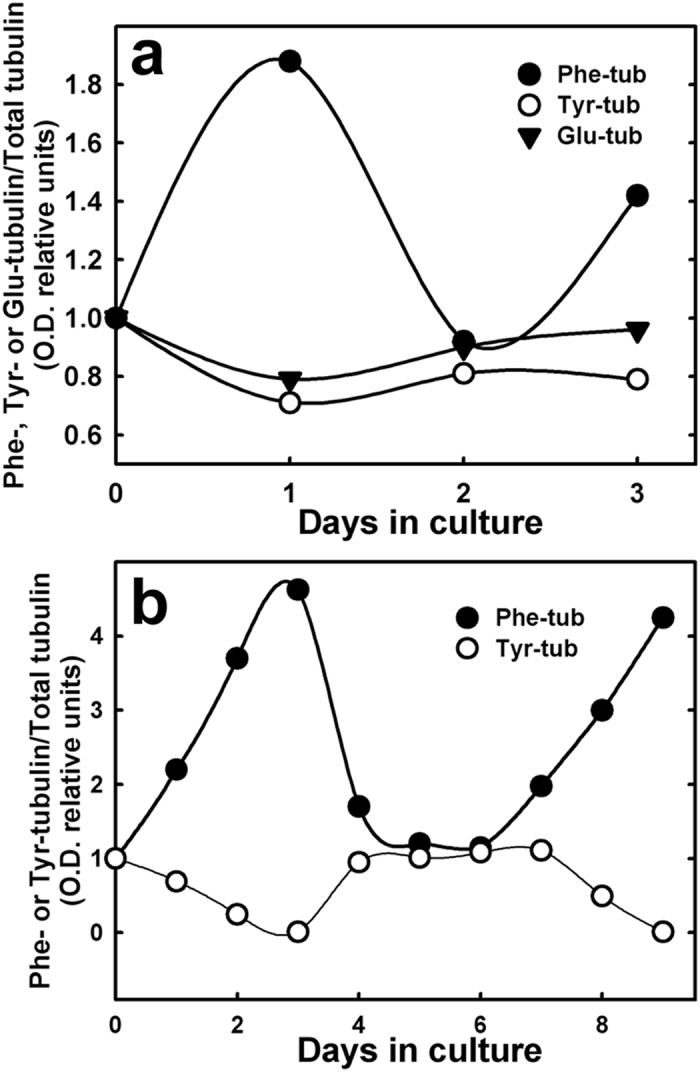
Post-translational incorporation of Phe into tubulin is reversible. (**a**) CAD cells were cultured in the alternating presence/absence of 4 mM Phe, starting with Phe (+) medium. At the end of day 1, medium was changed to Phe (−) medium. At the end of day 2, medium was changed to Phe (+) medium. Levels of Phe-, Tyr-, Glu-, and Total-tub were determined by Western blotting with specific antibodies on days 0, 1, 2, and 3. Optical density of each tubulin isospecies was standardized relative to Total-tub. (**b**) C6 cells were treated and analyzed as in (**a**), except that medium was changed at 2-day intervals.

To determine whether Phe incorporation into tubulin C-terminus was reversible, we cultured CAD cells in alternating day-by-day presence/absence of 4 mM Phe, with parallel Phe-free culture as control. Cells were collected each day and analyzed by Western blotting with anti-Phe-, anti-Tyr-, anti-Glu-, and anti-Total-tubulin antibodies. Quantitative analysis of bands following the initial 24 h in Phe (+) medium revealed significant Phe incorporation into the α-tubulin C-terminus, in place of Tyr (Fig. 2a). Subsequent 24-h incubation in Phe (−) medium led to reduction of Phe-tubulin and corresponding increases of Tyr- and Glu-tubulin. Subsequent 24-h incubation in Phe (+) medium led to a new Phe incorporation peak. These findings indicate that Phe entering the cell is incorporated into the α-tubulin C-terminus, and that this process is reversible. We obtained similar results using C6 glioma cells (Fig. 2b), suggesting that the reversibility of Phe incorporation is a general phenomenon.

### Phe-tubulin forms microtubules in CAD cells

**Figure 3:**
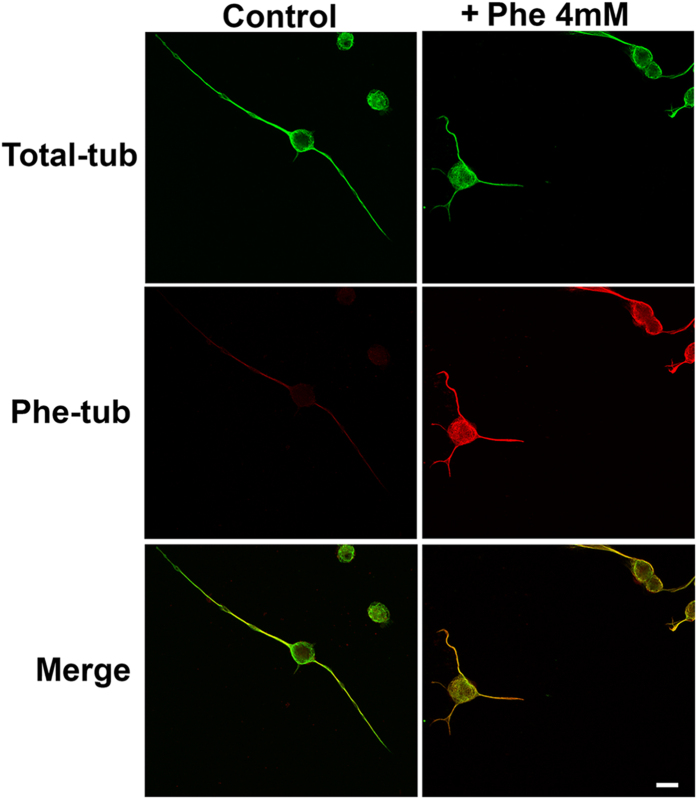
Phe-tubulin undergoes assembly into microtubules. CAD cells were cultured under differentiating conditions (absence of FBS) in the presence or absence of 4 mM Phe for 24 h, and then fixed with cold methanol. Cytoskeletons were immunolabeled with antibodies directed to Phe-tub (red staining) and Total-tub (green staining).

To investigate the possibility that Phe-tubulin can be polymerized into microtubules, we cultured CAD cells for several days under differentiating conditions in both 4 mM Phe (+) and (−) media. Cells were then fixed with cold methanol and subjected to double immunofluorescence analysis using antibodies against Phe-tubulin (red staining) and Total-tubulin (green staining). Confocal images showed that Phe (−) culture led to essentially no anti-Phe-tubulin antibody staining, whereas Phe (+) culture led to strong positive staining of the microtubule network (Fig. 3). Merge images showed complete co-localization of Phe-tubulin with Total-tubulin. These findings suggest that Phe-tubulin is able to form cytoskeletal microtubules without spatial preference in regard to other tubulin isospecies.

### Phe treatment slows down CAD cell proliferation without affecting cell viability

**Figure 4:**
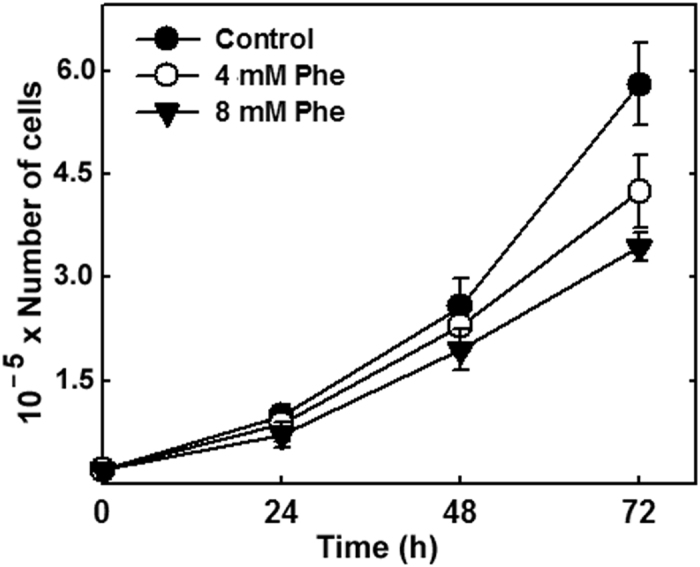
Effect of high Phe treatment on cell proliferation. CAD cells were seeded (2 × 10^4^ cells per 35-mm dish), incubated for 24, 48, or 72 h in the absence or presence of 4 mM or 8 mM Phe, stained with Trypan Blue, transferred to a Neubauer Chamber, and counted using an optical inverted microscope. Data shown are mean ± SD from three independent experiments.

The effects of Phe treatment on CAD cell viability and proliferation were evaluated by culturing cells in the absence or presence of 4 mM and 8 mM Phe, and measuring proliferation rate by Trypan Blue exclusion assay at 24, 48, and 72 h. Cell number under Phe (+) condition at both concentrations was lower than control value. This effect was evident at 48 h of culture and more pronounced at 72 h (Fig. 4), indicating that proliferation rate was reduced by high Phe concentration.

### High-concentration Phe treatment slows down neurite retraction and this effect is blocked by Tyr

**Figure 5:**
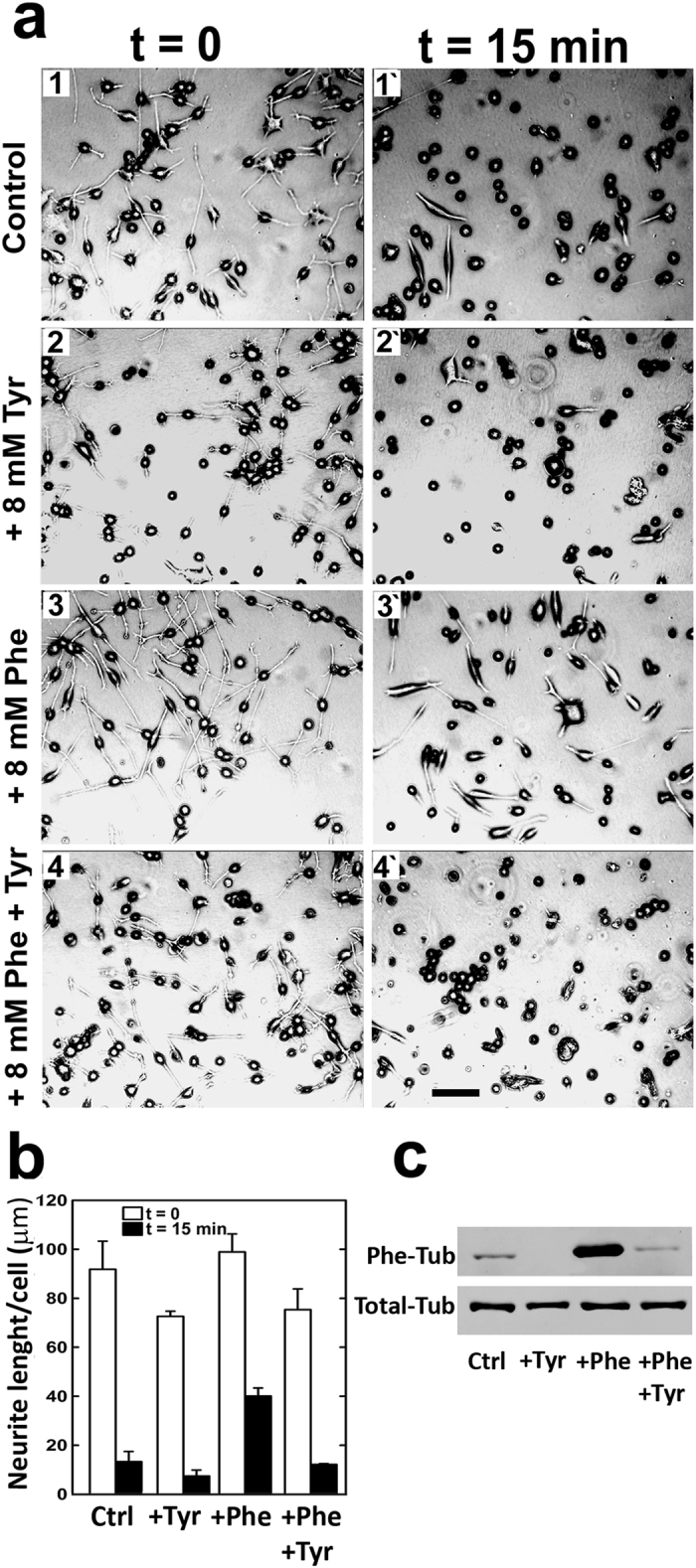
Effect of Phe treatment on neurite retraction. CAD cells were cultured 30 h in the absence or presence of 8 mM Phe under differentiating conditions. (**a**) At the end of this period but before addition of FBS (t = 0), phase contrast images were taken. FBS was then added to medium (final concentration 10%), and images were taken at t = 15 min. Total cell number counts and neurite lengths were obtained from the images. (**b**) For each treatment, the sum of all neurite lengths was divided by total cell number. Five or more randomly selected fields were counted for each time point. Data shown are mean ± SD. (**c**) Following a 15-min period of neurite retraction, cells were subjected to Western blotting with antibodies directed to Phe-tub and Total-tub. Blots shown are representative of three replicate experiments.

Because CAD cell proliferation rate was reduced by Phe treatment, we considered the possibility that this process also affects other cell properties; in particular, retraction of neurites in response to FBS. Cells cultured 30 h under differentiating conditions were treated with 8 mM Phe, 8 mM Tyr, a combination of the two amino acids, or vehicle alone. FBS was subsequently added to medium, and culture continued for 15 min. Photographs were taken at t = 0 and t = 15 min, and total cell numbers and neurite lengths were measured as described in Materials and Methods. Representative images are shown in Fig. 5a. In vehicle control (no Phe or Tyr added), clear neurite retraction was observed following FBS addition (**panels 1 and 1′**). Similar neurite retraction occurred in the 8 mM Tyr (+) group (**panels 2 and 2′**). Retraction was greatly inhibited in the 8 mM Phe (+) group (**panels 3 and 3′**). This inhibitory effect of 8 mM Phe on retraction was blocked by the co-presence of 8 mM Tyr (**panels 4 and 4′**). These findings were confirmed by quantitative analyses of neurite length under the above conditions. At t = 15 min after FBS addition, neurite length had decreased to 15% of initial value in the control group, but to 40% of initial value in the 8 mM Phe (+) group (Fig. 5b). Again, the inhibitory effect of Phe was blocked by Tyr; *i.e.*, retraction for the Phe/Tyr (+) group was similar to that for the control group (Fig. 5b). To evaluate the possible association of these observations with presence/absence of Phe in the α-tubulin C-terminus, we measured Phe-tubulin amounts at the end of the retraction process under the various experimental conditions. Phe-tubulin was only faintly present in the control group and the Phe/Tyr (+) group, but was abundant in the Phe (+) group (Fig. 5c). These findings suggest that inhibition of neurite retraction resulted from the presence of Phe in place of Tyr at the α-tubulin C-terminus in CAD cells.

### Phe treatment reduces microtubule dynamics

To evaluate possible involvement of microtubule dynamics in the inhibitory effect of high Phe on neurite retraction, we analyzed FRAP in GFP-tubulin-expressing CAD cells. In preliminary experiments, we found that treatment of transfected cells with 8 mM Phe resulted in Phe incorporation into C-termini of both, tubulin and GFP-tubulin (results not shown).

**Figure 6:**
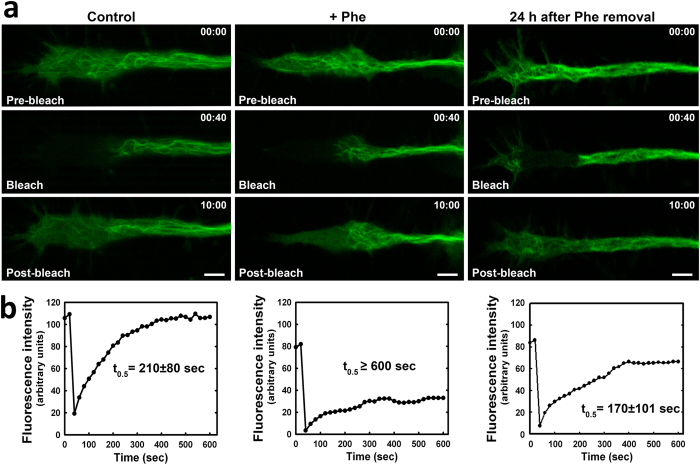
Effect of Phe treatment on microtubule dynamics. CAD cells were transfected with pEGFP-Tub and cultured 72 h under differentiating conditions in the absence (Control; left column) or presence (+Phe; middle column) of 8 mM Phe. A set of samples cultured in the presence of Phe during 72 h were subsequently incubated for an additional 24 h in the same culture medium without Phe (24 h after Phe removal; right column). Selected fields containing growth cones having no contact with other structures were subjected to FRAP analysis as described in Materials and Methods. Images were taken at 20-sec intervals during a 10-min period after bleaching. (**a**) Images are representative of three replicate experiments. (**b**) Quantification of fluorescence intensity. t_0.5_ values were calculated from four independent experiments.

Following 72 h differentiation of cells, selected areas of growth cones were photobleached and fluorescence recovery was measured as described in Materials and Methods. Fluorescence recovery half-life time (t_0.5_) in control cells was 210 ± 80 sec, indicating a high level of microtubule dynamics (Fig. 6; Control). In contrast, t_0.5_ in 8 mM Phe cells was >600 sec (Fig. 6; +Phe), indicating a strong reduction of microtubule dynamics. FRAP was also performed 24 h after removal of Phe from the culture medium (Fig. 6; 24 h after Phe removal) resulting in a t_0.5_ = 170 ± 101 sec indicating that microtubule dynamics at growth cones was recovered. When t_0.5_ was measured in growth cones of cells pre-treated with 10 μM taxol (a microtubule-stabilizing agent), no fluorescence recovery was observed after photobleaching of these cells, as expected (results not shown).

### Incorporation of Phe into the α-tubulin C-terminus results in reduction of microtubule dynamics

**Figure 7:**
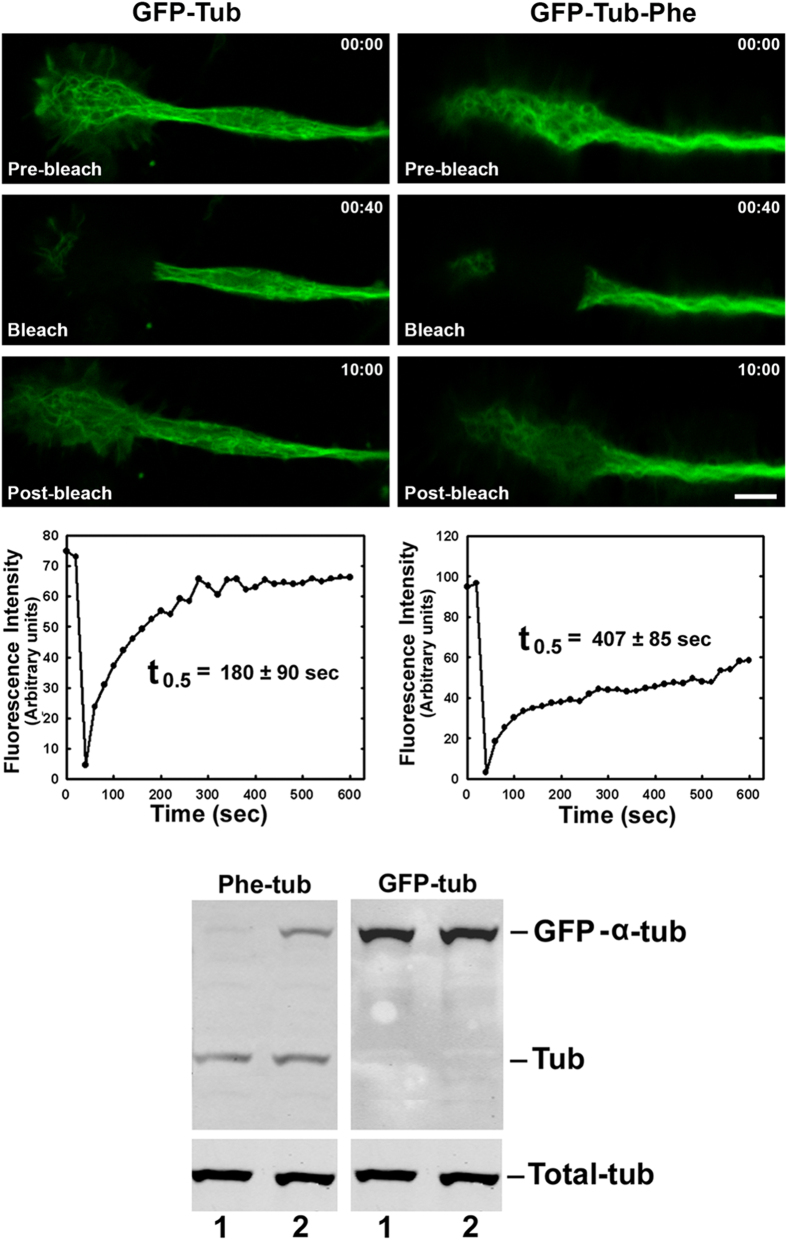
Effect of transfection of CAD cells with pEGFP-Tub-Phe or pEGFP-Tub on microtubule dynamics. CAD cells were transfected with pEGFP-Tub or pEGFP-Tub-Phe and cultured 72 h under differentiating conditions. Selected fields containing growth cones having no contact with other structures were subjected to FRAP analysis as described in Materials and Methods. Images were taken at 20-sec intervals during a 10-min period after bleaching. **Upper panel**: Images are representative of three replicate experiments. **Middle panel**: Quantification of fluorescence intensity. t_0.5_ values were expressed as mean ± S.D. from three independent experiments. **Lower panel**: Western blot analysis of cells transfected with pEGFP-tub (lanes 1) or with pEGP-tub-Phe (lanes 2). For a given amount of Total-tub (bottom), the two plasmids were similarly expressed (GFP-tub detected with antibodies to GFP), whereas GFP-tub-Phe was expressed only in cells transfected with pEGFP-tub-Phe (Phe-tub).

To determine whether the effect of Phe on microtubule dynamics was due to its incorporation into the α-tubulin C-terminus or to its involvement in other cellular events, previous to FRAP analysis cells were transfected with EGFP-Tub-Phe plasmid (encoding C-terminal Phe instead of Tyr), or pEGFP-Tub as control. The two plasmids had similar expression levels, and GFP-Tub-Phe was detected only when pEGFP-Tub-Phe was expressed (Fig. 7, lower panel). Under these conditions, FRAP analysis showed that t_0.5_ for cells expressing GFP-Tub-Phe was 407 ± 85 sec, whereas that of cells expressing GFP-Tub was 180 ± 90 sec (Fig. 7, upper and middle panels). Thus, microtubule dynamics was significatively reduced by transfection with plasmid containing GFP-α-tubulin-Phe, indicating that the presence of Phe-tubulin was responsible for the observed reduction of microtubule dynamics.

## Discussion

Generation of an antibody that specifically recognizes Phe at the α-tubulin C-terminus allowed us to study effects of this tubulin modification in living cells, *i.e.*, experimental cell culture systems. Building on previous reports of Phe incorporation into tubulin *in vitro* and *in vivo*^7^,^13^,^15^,^16^, we demonstrated occurrence of this post-translational modification in cells of neuronal (Figs 1 and 2) and glial origin (Fig. 2). When Phe concentration in culture medium was high, the Phe-tubulin isospecies accounted for ~40% of Total-tubulin, reflecting a significant change in tubulin tyrosination state. The reaction was reversible, and Phe-tubulin amount was a function of both Phe concentration in medium and incubation time. The ability of Phe-tubulin to undergo assembly into microtubules was demonstrated in differentiated CAD cells (Fig. 3), and cell viability was not altered by Phe incorporation. These findings and the fact that incorporation of Phe instead of Tyr is a minor, highly specific modification of “Tyr-tubulin” structure (*i.e.*, absence of hydroxyl group) might suggest that tubulin phenylalanination has no effect on cellular functions. However, cell proliferation rate was reduced by increased Phe concentration (Fig. 4). The degree of proliferation inhibition was positively correlated with Phe incubation time, possibly because of increasing Phe incorporation into tubulin (and therefore into microtubules) as a function of time (Fig. 1). Extrapolated to human brain development, this phenomenon could explain in part the elevated occurrence of microcephaly in babies from women with non-treated PKU^17^.

In CAD cells differentiated by culture in FBS-free medium supplemented with high-Phe (such cells contained a significant amount of Phe-tubulin as assessed by Western blotting), neurite retraction following FBS addition was slower than the rate for control cells (Fig. 5a,b). Such slowing of retraction rate was blocked by the presence of high-concentration Tyr (and related absence of Phe-tubulin; Fig. 5c), suggesting that the effect of high Phe in the absence of Tyr was due to Phe incorporation into tubulin.

Neurite retraction may depend in part on microtubule dynamics. We therefore used FRAP to evaluate the effect of Phe-tubulin on microtubule dynamics, and found that it was reduced in growth cones of CAD cells treated with high Phe (Fig. 6). When Phe-tubulin within cells was substantially reduced by 24 h incubation in medium without high Phe, dynamics returned to normal. The reduction of microtubule dynamics observed when cells were transfected with pEGFP-Tub-Phe strongly indicates that such reduction is due to the presence of Phe in the α-tubulin C-terminus. According to the “dynamic instability” theory, microtubule dynamics *in vitro* depends on the presence of GTP or GDP bound to tubulin at microtubule tips. On the other hand, dynamics in living cells is affected and regulated by numerous endogenous factors, including binding of MAPs, molecular motors, and plus-end tracking proteins (+Tips)^1^,^18^. Several post-translational modifications of tubulin (acetylation, glutamylation, glycylation) also influence microtubule dynamics^6^,^19^. Tubulin tyrosination state has not been reported to regulate dynamics; however, the present findings indicate that the enhanced level of an “anomalous” tubulin isospecies (Phe-tubulin) is involved in the reduction of proliferation rate, reduction of neurite retraction rate, and reduction of microtubule dynamics observed at growth cones. Increased levels of “Phe-microtubules” may alter interactions with the various proteins known to participate in regulation of microtubule functions.

Numerous cultured-cell and whole-brain studies addressing the molecular mechanisms that underlie neuronal and brain dysfunction in PKU patients have revealed alteration of various cellular functions by high-Phe treatment^20^,^21^,^22^,^23^. However, none of these studies has elucidated the pathological mechanism on a molecular basis. Our present findings clearly demonstrate that the presence of Phe at the α-tubulin C-terminus is associated with concomitant alterations of cell proliferation rate, microtubule dynamics, and neurite retraction. As an example, these results may provide an explanation of the observations of Horling *et al*.^24^ regarding reduced synaptic pruning in the hippocampus of a PKU mouse model in which neurotransmitter release is affected and neuronal activity is impaired. We cannot state definitively at this point that this tubulin modification is the primary cause of brain dysfunction in PKU patients. However, several observations are in line with this idea: (i) Direct participation of Phe in such a mechanism is confirmed by its post-translational incorporation into a protein *in vitro*^7^,^15^, *in vivo*^13^, and in cultured-cell systems (Fig. 1). (ii) Phe incorporation does not occur for any cellular protein other than tubulin, the primary constituent of microtubules. (iii) Phe-tubulin undergoes assembly into microtubules, resulting in formation of phenylalaninated microtubules (Fig. 3). (iv) The presence of Phe-tubulin has no effect on cell viability, but causes reduction of proliferation rate (Fig. 4). (v) Enhanced Phe-tubulin level results in reduction of microtubule dynamics and this effect is reverted by decreasing Phe-tubulin via incubation in the absence of high Phe (Fig. 6). (vi) Enhanced Phe-tubulin level inhibits neurite retraction (a process that depends on microtubule dynamics), whereas simultaneous high Tyr concentration blocks such inhibition by blocking Phe incorporation into tubulin (Fig. 5). (vii) Microtubules play essential roles in axonal transport of neurotransmitters, neuronal receptors, growth factors, lipids for membrane formation, and interactions with various soluble and membranous proteins^1^. Modification of any of these factors (*e.g.*, by Phe incorporation into tubulin) may therefore significantly alter neuronal functions and brain development.

In conclusion, since the described experiments were performed with mouse neurons, it is clear that our results do not permit us to firmly state that the pathogenesis of PKU brain dysfunction in human being is caused by the modification of tubulin. However, this idea cannot be obviated. In fact, our findings are compatible with the hypothesis that Phe incorporation into the α-tubulin C-terminus could be the first event (or among the initial events) in the molecular pathways leading to the neural and brain dysfunctions that characterize PKU. Beyond the scope of the present work, further experiments addressing the mechanism on the eventual relationship between Phe incorporation into tubulin and PKU disease are in progress.

## Materials and Methods

### Chemicals

Chemicals, antibodies, and culture media were from Sigma-Aldrich (St. Louis, MO, USA) unless stated otherwise. Rabbit polyclonal antibody specific to Glu-tubulin was prepared in our laboratory as described by Gundersen *et al*.^25^. IRDye 800CW goat anti-mouse IgG and IRDye 800CW goat anti-rabbit IgG were from Li-Cor Biosciences (Lincoln, NE, USA). Lipofectamine 2000 was from Invitrogen (Grand Island, NY, USA). Fetal bovine serum (FBS) was from Natocor (Córdoba, Argentina). Y-27632 and FluorSave were from Calbiochem (Billerica, MA, USA).

### Cell culture

HeLa and C6 cells were cultured in DMEM, and CAD cells were cultured in DMEM/F12 (50:50, v/v); both media were supplemented with 10% (v/v) FBS, 10 units·mL^−1^ penicillin, and 100 μg·mL^−1^ streptomycin. All cultured cells were maintained at 37 °C in humidified 5% CO_2_ atmosphere. Differentiation of CAD cells was induced by replacement of medium with FBS-free medium, and differentiation status (neurite growth) was assessed by optical microscopic examination. In some experiments, cells were incubated in the presence or absence of various concentrations of Phe and/or Tyr. Because Tyr solubility in water is limited, a stock solution of Tyr (230 mM) was prepared in 1 M HCl. An aliquot of this solution was added to culture medium to obtain the desired Tyr concentration, pH was immediately adjusted to 7.5 using 1 M NaOH, and the modified medium was added to cells.

### Cell viability and proliferation

Trypan Blue exclusion assay was used for counting of living cells and monitoring of cell proliferation. CAD cells were seeded in 35-mm dishes (2 × 10^4^ cells/dish) and incubated for 24, 48, or 72 h in the absence or presence of 4 mM or 8 mM Phe. For the assay, Trypan Blue solution was added to cell culture (final concentration 0.75%) at room temperature for 3 min, cells were transferred to a Neubauer Chamber, and living and dead cells were counted using an optical inverted microscope (model Axiovert 135, Carl Zeiss; Jena, Germany).

### Neurite length

Microphotographs taken during experiments were examined to determine neurite length. For each time point and treatment, five areas were randomly selected and analyzed using the Fiji software program (National Institutes of Health; Bethesda, MD, USA). Neurite lengths were measured, and the sum of all lengths was divided by the total number of cells in the given area. Images were taken by the Axiovert 135 inverted microscope equipped with an Olympus XM10 camera and cell Sens Digital Imaging software (Olympus; Center Valley, PA, USA). Results (mean ± SD from three independent experiments) were expressed as neurite length (μm)/cell.

### Immunofluorescence

Cells were cultured on coverslips, fixed with cold (−20 °C) methanol for 10 min, rehydrated, washed with NaCl/P_i_, incubated with 5% (w/v) BSA in NaCl/P_i_ for 1 h, incubated with primary antibody anti-Phe (1:1000) or anti-Total-tubulin (1:1000) for 2 h at 37 °C, washed three times with NaCl/P_i_, and incubated for 1 h at 37 °C with Alexa Fluor 488 goat anti-mouse IgG (1:2000) and Alexa Fluor 594 goat anti-rabbit IgG (1:2000). Coverslips were mounted in FluorSave, and fluorescence was observed by confocal microscopy (model FluoView 1000, Olympus). When comparison of different preparations was necessary, photographs were taken with the same gain value.

### Fluorescence recovery after photobleaching (FRAP)

Undifferentiated CAD cells were grown to 80% confluence in a microslide glass-bottom chamber (Ibidi; Martinsried, Germany), transfected with pEGFP-Tub (Clontech; Mountain View, CA, USA) according to the manufacturer’s protocol, grown for 72 h under differentiating conditions, and subjected to FRAP analysis, *i.e.*, photobleaching of GFP-tubulin or GFP-tubulin-Phe in a defined area of the cell followed by measurement of fluorescence recovery in this area as a function of time. As a control, fluorescence values were also measured in a background region. For FRAP experiments, 5 to 7 growth cones per condition and experiment were analyzed. Before and after photobleaching images were acquired every 20 sec during a 10-min period with a 20 × 0.5 numerical aperture objective (Plan-Neofluar, Carl Zeiss), with the confocal pinhole of the microscope fully open. Selective photobleaching of GFP was performed under a laser-scanning confocal microscope (FluoView 1000, Olympus). During image acquisition, living cells were maintained at 37 °C in 5% CO_2_ atmosphere. Fluorescence in selected regions of interest was quantified using the Fiji program.

### Introduction of mutations into pEGFP-Tub

As starting point for mutagenesis, we used full-length pEGFP-Tub as a template. Point mutation (α-tubulin C-terminal Tyr to Phe) was introduced using the QuikChange Site-directed mutagenesis kit (Agilent Technologies; Santa Clara, CA, USA). PCR reactions were performed using the plasmid and mutagenic primers GTTCACCTCCCTGCTCATGGAACG and CGGTCTAGATTAGAATTCCTCTCCTTCTTCCTCACCCTC (Invitrogen). Colonies positive for the desired mutation were grown under standard conditions in LB broth, and plasmids were isolated using a plasmid DNA purification kit (Sigma-Aldrich). All mutations were verified by DNA sequencing.

### SDS-PAGE and Western blotting

Proteins were separated by SDS-PAGE (10% gels) and transferred to nitrocellulose sheets. The sheets were blocked with 5% nonfat dried milk powder in NaCl/P_i_, incubated with primary antibodies directed to Total-, Tyr-, Glu-, or Phe-tubulin (1:1000) for 2 h at room temperature in 1% nonfat dried milk powder in NaCl/P_i_, washed three times, incubated with infrared fluorescent secondary antibodies for 1 h at room temperature (1:25000), washed, and scanned with an Odyssey infrared scanner (Li-Cor). Optical density of bands was quantified using the Scion Image software program (Scion Corp, USA).

### Microscopy facilities

All microscopic observations, including those for FRAP analysis, were performed at the facilities of the Centro de Microscopía Óptica y Confocal de Avanzada (CIQUIBIC-CONICET; Córdoba, Argentina).

## Additional Information

**How to cite this article**: Ditamo, Y. *et al*. Post-Translational Incorporation of L-Phenylalanine into the C-Terminus of α-Tubulin as a Possible Cause of Neuronal Dysfunction. *Sci. Rep.* **6**, 38140; doi: 10.1038/srep38140 (2016).

**Publisher's note:** Springer Nature remains neutral with regard to jurisdictional claims in published maps and institutional affiliations.

## Supplementary Material

Supplementary Information
