# Supplementary material for: Post-Translational Incorporation of L-Phenylalanine into the C-Terminus of α-Tubulin as a Possible Cause of Neuronal Dysfunction
Source: Sci Rep. 2016 Dec 1;6:38140. doi: 10.1038/srep38140 (PMC5131269; doi:10.1038/srep38140)
Supplement: Supplementary Information [file srep38140-s1.doc]

**Supplementary Information**

**POST-TRANSLATIONAL INCORPORATION OF L-PHENYLALANINE INTO THE C-TERMINUS OF α-TUBULIN AS A POSSIBLE CAUSE OF NEURONAL DYSFUNCTION**

Yanina Ditamo, Yanela M. Dentesano, Silvia A. Purro, Carlos A. Arce, and C. Gastón Bisig *

**Centro de Investigaciones en Química Biológica de Córdoba, CIQUIBIC, CONICET, and Departamento de Química Biológica, Facultad de Ciencias Químicas, Universidad Nacional de Córdoba, Ciudad Universitaria, X5000HUA, Córdoba, Argentina.**

**Supplementary Methods**

**Preparation of soluble rat brain extract and purification of tubulin.** Brains from 15- to 30-day-old Wistar rats were homogenized in 1 vol MEM buffer (100 mM MES buffer adjusted with NaOH to pH 6.7, containing 1 mM EGTA, 1 mM MgCl_2_, and a mixture of protease inhibitors; Sigma). The homogenate was centrifuged at 100,000 x *g* for 1 h at 2-4°C,and supernatant solution was collected and used immediately. For purification of tubulin, 1x cycled microtubule protein was chromatographed on a Mono-Q column (GE Healthcare; Pittsburgh, PA, USA) as described previously[^1^](#_ENREF_1).

**Generation of a specific Phe-tubulin antibody.** The immunization protocol was similar to that we used previously for production of antisera specific to 3-nitro-Tyr-tubulin and to azatyrosine-tubulin . In brief, Gly-Glu-Glu-Phe peptide (C-terminus of α-tubulin with Tyr replaced by Phe) was bound through its amino group to KLH using glutaraldehyde as a crosslinker. The resulting protein (500 µg) was mixed with complete Freund’s adjuvant (1:1, v/v) and used for the primary injection. Subsequent booster immunizations were performed every 15 days, using 500 µg of the same protein preparation emulsified in incomplete adjuvant. Blood was collected 15 days after each injection, and sera were tested for affinity and specificity.

**Specificity of polyclonal antibody.** Freshly prepared soluble rat brain extract was treated with 10 µg/mL pancreatic carboxypeptidase A (CPA) for 30 min at 37 ºC and then passed through a Sephadex G-25-80 column equilibrated with MEM buffer to eliminate free amino acids. CPA was inactivated by 50 µg/mL of CPA inhibitor (CPI). Aliquots of the resulting preparation were incubated 30 min at 37ºC with 1 mM Tyr or 1 mM Phe under incorporating conditions (per mL incubating medium: 0.9 mL soluble brain extract, 2.5 µmol ATP, 12.5 µmol MgCl_2_, 30 µmol KCl, 100 µmol MES buffer, pH 6.7). When incubation was completed, Laemmli sample buffer was added, and samples were immunoblotted and stained with antibodies directed to Total-, Tyr-, or Phe-tubulin (1:1000).

**Quantification of Phe-tubulin.** Absolute amounts of Phe-tubulin were measured using cultured HeLa cells, which contain only Tyr-tubulin and no Glu-tubulin.Total-tubulin amount was determined by comparison with pure tubulin standard. These data provided a useful Tyr-tubulin standard and were used to obtain a standard curve of optical density as a function of ng Tyr-tubulin. A standard curve for Glu-tubulin was obtained by treating HeLa cell tubulin with CPA, which transformed all Tyr-tubulin to Glu-tubulin.

**Supplementary Results**

**Supplementary Figure 1: Specificity of anti-Phe-tubulin antibody.** Soluble rat brain extract (SN, stained with Coomassie Blue) was treated with CPA and then with the CPA inhibitor CPI. Two separate aliquots were incubated under conditions for incorporation of Tyr (lanes 1) or Phe (lanes 2) into the C-terminus of α-tubulin, and then subjected to Western blotting and immunostaining with antibodies directed to Total-tub, Tyr-Tub, or Phe-Tub. For the two right-hand blots, anti-Phe-tubulin antibody was incubated 1 h at 25 ºC in the presence of 500 µM Tyr or 500 µM Phe.


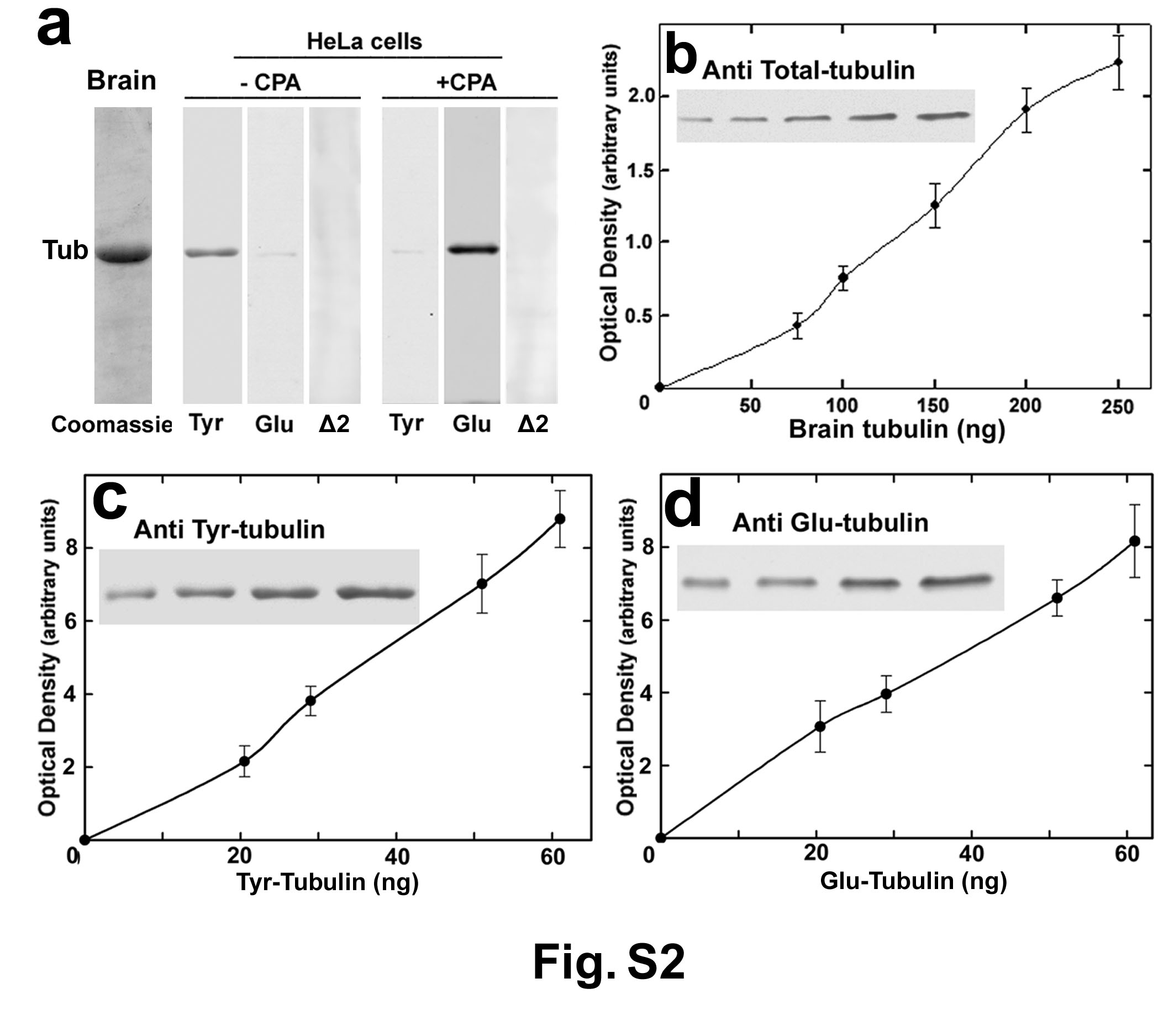


**Supplementary Figure 2: Standard curves for quantification of Total-, Tyr- and Glu-tub:** (**a**) Purified rat brain tubulin was subjected to SDS-PAGE (10% acrylamide) and stained with Coomassie Brilliant Blue (left). HeLa cells were harvested, pelleted by centrifugation, resuspended in MEM buffer, and sonicated. After centrifugation at 100,000 x *g* for 30 min at 2-4ºC, aliquots of the supernatant fraction were incubated in the absence or presence of 10 μg/mL CPA for 15 min, and inactivated by dilution with 1x Laemmli sample buffer and heating at 90ºC for 5 min. Samples were subjected to Western blotting and stained with antibodies to Tyr-, Glu-, and Δ2-tubulin. (**b**) The indicated amounts of purified rat brain tubulin were subjected to Western blotting and stained with anti-Total-tubulin antibody. (**c**) The indicated amounts of HeLa cell tubulin (not treated with CPA), representing equivalent amounts of the Tyr-tubulin isospecies, were subjected to Western blotting and stained with anti-Tyr-tubulin antibody. (**d**) The indicated amounts of HeLa cell tubulin (treated with CPA), representing equivalent amounts of the Glu-tubulin isospecies, were subjected to Western blotting and stained with anti-Glu-tubulin antibody. Optical densities of all bands were measured. Only optical density values within the linear range are shown.

**Supplementary Table 1. Quantification of Phe-tubulin in CAD cell extracts.**

By using curves from Supplementary Fig. 2, we determined by Western blotting the amounts of Tyr-, Glu- and Total-tubulin in CAD cells previously incubated for 48 h in the absence or presence of 4 mM Phe. The amount of Phe-tubulin was estimated from the difference between Total-tubulin minus the sum of Tyr- plus Glu-tubulin. Results are expressed in ng and the percentage of Phe-tubulin is shown in parentheses. Results are from 3 independent experiments.

Total-tub Tyr-tub Glu-tub Phe-tub

__________________

ng ng ng ng (%)

(A) (B) (C) A-(B+C)

_______________________________________________________________

No Phe 104±7 79±5 20±3 5±1 (4%±1)

+ 4 mM Phe,

t = 48 h 106±8 44±4 13±2 49±4 (46%±4)

**Supplementary Information References**

1. Carbajal A, Chesta ME, Bisig CG, Arce CA. A novel method for purification of polymerizable tubulin with a high content of the acetylated isotype. *The Biochemical journal* 449, 643-648 (2013).

2. Bisig CG, Purro SA, Contin MA, Barra HS, Arce CA. Incorporation of 3-nitrotyrosine into the C-terminus of alpha-tubulin is reversible and not detrimental to dividing cells. *European journal of biochemistry / FEBS* 269, 5037-5045 (2002).

3. Purro SA, Bisig CG, Contin MA, Barra HS, Arce CA. Post-translational incorporation of the antiproliferative agent azatyrosine into the C-terminus of alpha-tubulin. *The Biochemical journal* 375, 121-129 (2003).
